# A Three-Step, Gram-Scale Synthesis of Hydroxytyrosol, Hydroxytyrosol Acetate, and 3,4-Dihydroxyphenylglycol

**DOI:** 10.3390/molecules24183239

**Published:** 2019-09-05

**Authors:** Amalia D. Kalampaliki, Vassiliki Giannouli, Alexios-Leandros Skaltsounis, Ioannis K. Kostakis

**Affiliations:** 1 Division of Pharmaceutical Chemistry, Department of Pharmacy, National and Kapodistrian University of Athens, Panepistimiopolis-Zografou, 15771 Athens, Greece; 2 Division of Pharmacognosy & Natural Product Chemistry, Department of Pharmacy, National and Kapodistrian University of Athens, Panepistimiopolis-Zografou, 15771 Athens, Greece

**Keywords:** hydroxytyrosol, hydroxytyrosol acetate, 3,4-dihydroxyphenylglycol, large scale synthesis, microwave irradiation, nutraceutical

## Abstract

Hydroxytyrosol and two other polyphenols of olive tree, hydroxytyrosol acetate and 3,4-dihydroxyphenylglycol, are known for a wide range of beneficial activities in human health and prevention from diseases. The inability to isolate high, pure amounts of these natural compounds and the difficult and laborious procedures for the synthesis of them led us to describe herein an efficient, easy, cheap, and scaling up synthetic procedure, from catechol, via microwave irradiation.

## 1. Introduction

**Figure 1:**
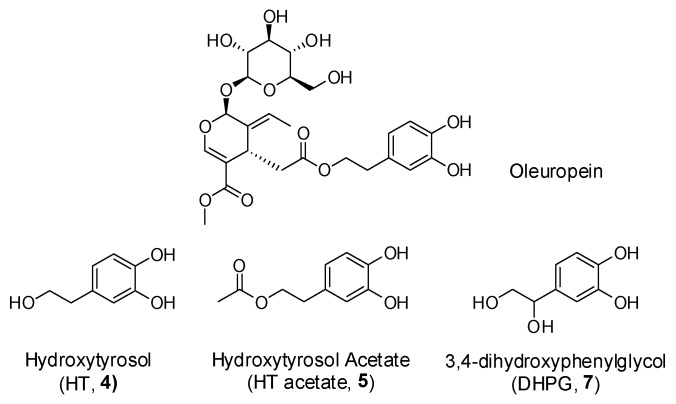
Major polyphenols of olive tree.

Polyphenols are a wide family of compounds found in plant-based foods, such as cranberries, grapes, olives, and walnuts, and have many diverse biological activities. One of the most important components of the polyphenol family is hydroxytyrosol (HT), a simple o-diphenol found in leaves and fruits of olive tree, virgin olive oil, as well as its wastewaters. It is derived from the hydrolysis of oleuropein, during the maturation of olives, storage of oil, and preparation of table olives. Two other important polyphenols of olive tree are hydroxytyrosol acetate and 3,4-dihydroxyphenylglycol (DHPG) (Figure 1).

The high interest concerning these compounds focuses on their involvement in many aspects of human health [1,2,3]. HT function as an efficient free radical scavenger [4,5,6,7,8] and metal ion chelator, such as Cu and Fe, which catalyze free radical generation, to lower hydroxyl radical formation [9]. This activity can be ascribed to the electron donating ability of the o-hydroxyl groups and subsequent formation of intramolecular hydrogen bonds with free radicals [10,11]. HT effectively permeates cell membranes via passive diffusion mechanisms and counteracts the cytotoxic effects of free radicals, which are formed as a result of metabolic processes in the organism [12].

There are clear epidemiological and biochemical evidence indicating that HT possesses a cardioprotective effect, reducing the risk of coronary heart disease and atherosclerosis, through inhibition of the LDL oxidation and platelet aggregation [13,14,15,16,17]. The European Food Safety Authority (EFSA) has endorsed the claim that consumption of olive oil polyphenols (standardized by the content of HT and its derivatives) contributes to the protection of blood lipids from oxidative damage. The panel considers that in order to bear the claim, 5 mg of HT and its derivatives should be consumed daily [18,19]. Additionally, during 2017, EFSA also confirmed that synthetic HT can be added in formulas for supplementary dietary with no difference from the natural compound [20].

Accumulating evidence associate HT with protection against the development of aging, which is intimately connected with its antioxidant [21] and anti-inflammatory activity [22,23]. Additionally, HT exerts anticancer effects through the activation of molecular signaling pathways leading to apoptosis and growth arrest in several tumor cell lines in vitro [4,5,24]. Furthermore, evidence has shown that HT interacts with pro-angiogenic growth factors, i.e., VEGF, exerting its potential anti-angiogenic activity [25,26].

Due to the implication of oxidative stress and generation of reactive oxygen species in the aggregation of beta-amyloid peptide and tau protein, it is proposed that HT may offer a protective or therapeutic alternative against Alzheimer’s disease [27] and other neurodegenerative diseases, such as Huntington’s Disease [28], multiple scleroses [29], and Parkinson’s Disease [30].

On the other hand, many findings suggest that HT protects against macular degeneration, reducing the oxidative stress in the epithelial cells of the retina, occurred by the accumulation of acrolein [31,32]. Moreover, this polyphenol seems to interact with the formation of bones and the preservation of the bone mineral density, resulting in positive effects against osteoporosis and bone loss [33]. It is imperative to notice that HT is non-genotoxic and non-mutagenic even at very high doses [34,35,36], and a no observed adverse effects level (NOAEL) of 500 mg/kg/day has been reported [37].

Regarding HT acetate, its antioxidant activity is much higher than that of a-tocopherol and oleuropein, but similar to HT [38]. Thus, the applications of HT could be extended using the lipophilic HT acetate which shows good solubility in oils and emulsions and could be used for controlled release of HT when needed, through its hydrolysis from lipases [39,40]. Additionally, DHPG has potent antioxidant [41,42] and antimicrobial activity [43,44], however, there are limited studies regarding its isolation from olive tree and its activity, probably because this polyphenol is water soluble and extremely unstable under alkali treatment.

Due to the variety of the biological activities and excellent safety profile, HT, HT acetate, and DHPG are currently three of the most actively investigated natural polyphenols and could be essential supplements in the pharmaceutical and nutraceutical industry for the treatment and prevention of cardiovascular diseases, reduction of hypertension, carcinogenesis, atherosclerosis, and many other uses [45]. Additionally, HT and HT acetate could be used in the dermocosmetic industry for the protection of skin from oxidative stress or used as a preservative in the food industry [46].

## 2. Results and Discussion

The great interest of this high-added value natural compound triggered the development of various protocols in order to recover HT from olive industry wastewaters, but usually, it is isolated as a mixture with other phenolic compounds. Additionally, a few procedures describe the isolation of HT through the hydrolysis of oleuropein, a natural compound found mainly in olive tree leaves and other sources such as *Ligustrum Vulgare* [47,48,49,50,51]. Nevertheless, these approaches, although eco-friendly, are costly, often require the use of harmful solvents in extensive amount and usually result in low yield.

**Figure 2:**
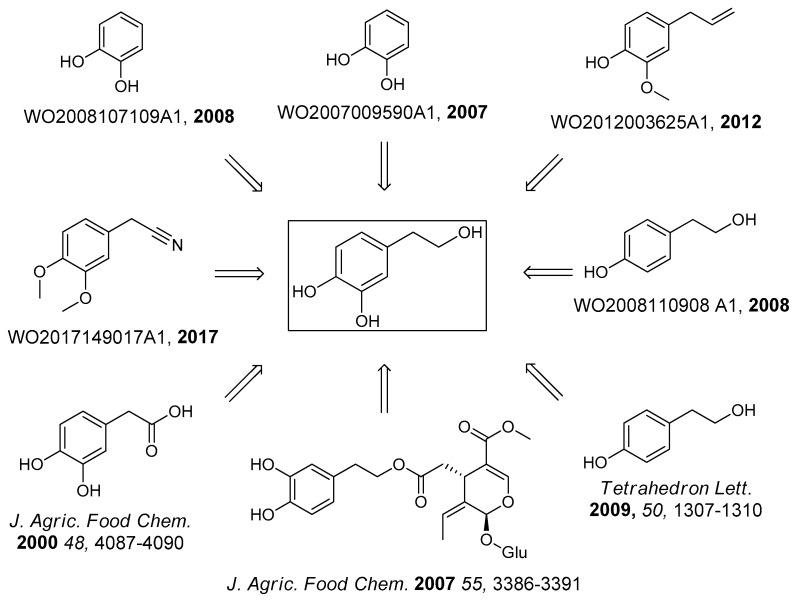
Synthetic approaches for hydroxytyrosol.

Due to the described problems, there has been a growing interest in developing an efficient synthetic route in order to obtain pure HT, but the synthesis is problematic since this compound is relatively soluble in water and polar organic solvents, therefore, the complete isolation through extraction from the reaction media is difficult [52]. Furthermore, HT is easily oxidized and relatively unstable during silica gel purifications and long-term storage [52]; thus adequate precautions must be taken (acid pH and absence of oxidants are crucial), concerning the synthesis and purification. Numerous synthetic approaches (more than 85 references in Reaxys database and 18 patents) [46,47,48,50,51,52,53,54,55,56,57,58,59,60,61,62,63,64,65,66,67,68,69] for the synthesis of HT have been described; however all these methods appear to have many disadvantages, i.e., low yields, laborious purifications, high cost, and many byproducts (Figure 2). The starting material for most of these synthetic procedures is tyrosol, through an oxidative procedure [51,57,58,59,62,64,69], though, the major disadvantage of the abovementioned approaches consists mainly of the use of non-eco-friendly oxidative reactants and the relatively high price of tyrosol. Alternatively, the synthesis of HT has been presented by the reduction of 3,4-dihydroxyphenylacetic acid but the commercial price of the acid (or the price of the corresponding nitrile) is too high [48,52,60,66]. Additionally, the isolation of HT from the reaction media is difficult (because of the water solubility), thus the overall yield is often low. Interestingly, only three synthetic approaches use catechol as starting material, even though this molecule is 6400-fold cheaper than tyrosol (Sigma-Aldrich 2019) (Table 1).

Concerning the synthesis of HT acetate, several synthetic procedures have been described, such as selective transesterification of the primary hydroxyl using suitable Lewis acids or protection of the phenolic hydroxyls before the acetylation [38,46,52,57,58,59,62,64,67,68,69,70,71,72,73]. Nevertheless, all of the above procedures are problematic since they use tyrosol or HT as staring material, two expensive compounds. Also, referring to the protection/deprotection approach, many steps are involved, thus the overall yield is relatively low. Regarding the synthesis of DHPG, only one synthetic procedure has been described with many steps, low yield, and vigorous conditions [74].

Prompted by the above, herein we described a simple and efficient synthesis of HT, with inexpensive catechol as staring material, without using extreme experimental conditions or laborious purifications. Additionally, the synthesis of HT acetate and DHPG was illustrated by engaging an intermediate from the procedure of HT synthesis.

**Scheme 1:**
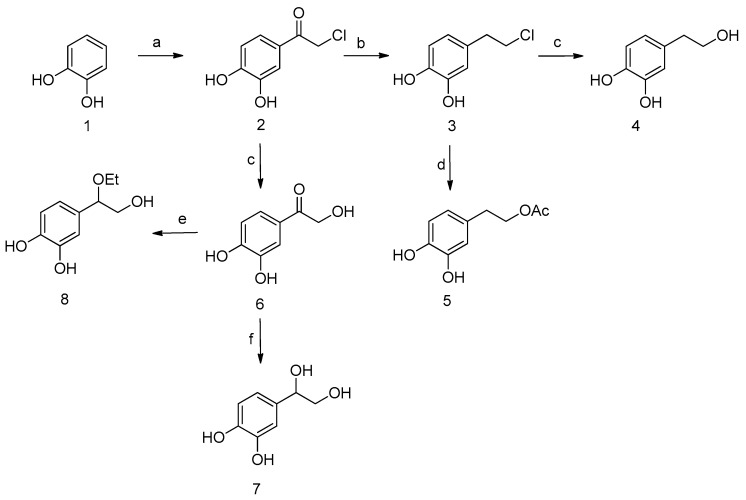
Reagents and conditions: (**a**) chloroacetyl chloride, POCl_3_, 105 °C, 150 W; (**b**) Et_3_SiH, CF_3_CO_2_H or BF_3_.Et_2_O, 25 °C; (**c**) H_2_O, 100 °C, 100W; (**d**) CH_3_CO_2_K, DMF dry, 70 °C; (**e**) H_2_, Pd/C, HClO_4_, abs. ethanol; (**f**) H_2_, Pd/C, methanol.

To overcome the solubility and stability limitations of HT, we envisioned a three-step synthetic procedure depicted in Scheme 1. The first step of the synthesis involved Friedel–Craft acylation of catechol (**1**) with chloroacetyl chloride in nearly quantitative yield. The preparation of chloride **2** has been previously reported through many different procedures [75,76]; however, the method reported herein, via microwave irradiation, was straightforward, simple, high yielding, and cost-effective. Thus, after vacuum evaporation of the excess of POCl_3_ and trituration with water, the crude product was used without any further purification. It should be noted that the evaporation of the excess of POCl_3_ before the trituration with water was crucial, otherwise, and if the reaction mixture is poured into ice-water (an experimental usually used for reactions with POCl_3_), the overall yield is remarkably reduced (about 40%) even after extraction of the reaction media.

The next step concerns the reduction of the carbonyl group; still all the initial attempts, under several conditions, provided complex mixtures. Among the variety of conditions used for the reduction of compound **2**, only the reduction with triethylsilane in the presence of a Lewis acid gave the desired chloro compound **3** [77,78,79,80]. More precisely, of the various Lewis acids and reaction conditions that were tested, the use of a fourfold excess of trifluoroacetic acid or a fivefold excess of boron trifluoride etherate and a twofold excess of triethylsilane derived the desirable catechol **3** in 95% or 92%, respectively. The reaction proceeded smoothly with no formation of byproducts. It is remarkable to note that the crude of the reaction was readily purified, after vacuum evaporation of the volatiles, by just trituration with boiling cyclohexane and vacuum evaporation of the solvent.

Finally, chloride **3** was readily converted to the corresponding HT (**4**) upon hydrolysis with distilled water, via microwave heating in a Milestone Start E apparatus. Reaction conditions were optimized, varying applied power and temperature, measured by fiber optic contact thermometer. The best results were obtained using programmed irradiation at 700 watts, at 101 °C, at open vessel conditions, and HT was obtained in >92% yield.

It is important to note that by the above-mentioned conditions, because of the absence of basic media (actually the reaction media was acidic due to the hydrochloric acid produced by the reaction) or oxidants, the reaction proceeded readily. Furthermore, the isolation was very simple, and no chromatography purification was needed. After completion of the reaction, the mixture was extracted with dichloromethane, and the aqueous phase was vacuum evaporated at low temperature or lyophilized, in order to obtain pure HT. Scaling up to 10 g of the above-mentioned synthetic procedure afforded reproducible overall yields. Higher scale-ups, up to 120 g showed the same excellent results only by modifying the time reaction conditions. The final step of the hydrolysis of chloride **3** to HT (**4**) was also accomplished via conventional heating, but much more time was needed. In particular, the hydrolysis of 23 g of chloride **3** to HT **(4)** required 4 h, instead of 32 h of conventional heating. Additionally, the yield of the reaction was lower (65% vs. 94.8% by MW heating) probably due to the instability of HT. Most importantly, the purification of HT, synthesized by MW-assisted hydrolysis, was very simple allowing the overall workup simplification, whilst the same purification of the product, of the conventional heating procedure, was laborious.

The overall yield of proposed approach was up to 85%, with only three steps involved, whilst the purification was easy with no silica gel chromatography needed (Table 1). Nevertheless, the main advantage of the procedure concerned the use of catechol as staring material, an inexpensive compound, instead of tyrosol, which is usually used, and the reproducibility of the method, even in scale-up conditions. There are only three other cases in which catechol is used as starting material, though, the developed approach possessed the highest yield, with only three steps involved. Besides, only limited amount of solvents, for the reaction and the purification procedures, were needed.

Taking advantage of the intermediate **3**, we were also able to prepare HT acetate (**5**), via a one-step reaction, upon treatment with potassium acetate in dry DMF at 70 °C, which yielded up to 85% (Scheme 1). It is important to notice that the presented procedure was straightforward, with no protection of the phenolic groups needed. The overall yield of the procedure was up to 73%, with minimum purifications, since the only silica gel column chromatography was performed on the final step. Into the best of our knowledge, this was the only approach that used catechol for the synthesis of HT-acetate, since all the other procedures use tyrosol or HT as starting material (Table 1). In addition, we can assert that the presented procedure for the synthesis of HT acetate could be used for the synthesis of various HT lipophilic esters, in order to study their biological activity.

Regarding the synthesis of 3,4-dihydroxyphenylglycol (**7**), a slightly different methodology was developed, which involved the initial hydrolysis of compound **2** to provide alcohol **6**. Although this hydrolysis is already described in the literature via HCOONa/HCOOH system [40], in our hands proceeded almost quantitively, by microwave heating, in distilled water. The reaction was scalable up to grams, without any by-products formed, while the purification was very simple, by trituration with ethyl acetate. Finally, target DHPG (**7**) was prepared through catalytic hydrogenation in methanol, in an 82% overall yield. The overall yield of the proposed procedure was up to 70%, far better than the previous procedure, with only three steps involved (Table 1) [74]. Interestingly, reduction of keto compound **6** over palladium on activated carbon, in the presence of a few drops of perchloric or hydrochloric acid, in abs. ethanol, afforded ether **8** [81], presumably through protonation of the intermediate alcohol and subsequent nucleophilic substitution by ethanol. It should be noted that this compound, even as a racemic mixture, is very interesting, since it is a stable form of DHPG, with better lipophilicity, and could be useful to study the therapeutic role and potentiality of DHPG.

## 3. Materials and Methods

### General Experimental Procedures

All chemicals were purchased from Alfa Aesar (Ward Hill, MA, USA). Melting points were determined on a Büchi apparatus and were uncorrected. ^1^H NMR, ^13^C NMR and 2D spectra were recorded on a Bruker Avance III 600 spectrometer (Bruker BioSpin GmbH, Rheinstetten, Germany), in deuterated solvents and were referenced to TMS (δ scale). The signals of ^1^H and ^13^C spectra were unambiguously assigned by using 2D NMR techniques: ^1^H^1^H COSY, HMQC, and HMBC. Flash chromatography was performed on Merck silica gel 60 (0.040–0.063 mm). Analytical thin layer chromatography (TLC) was carried out on pre-coated (0.25 mm) Merck silica gel F-254 plates (Merck KGa, Darmstadt, Germany). Mass spectra were recorded with a QqToF Premier MicroMass MS technology (Waters, Manchester, UK). The purity of all the synthesized compounds was >95%, determined by ^1^H NMR. Microwave experiments were performed on Milestone Start E apparatus (Milestone Srl, Bergamo, Italy). ^1^H and ^13^C-NMR spectra are available online as Supplementary Materials. For a schematic overview and detailed steps, see Scheme 1.

2-chloro-1-(3,4-dihydroxyphenyl)ethanone (**2**): A solution of catechol **1** (100 g, 0.94 mol) and chloroacetyl chloride (84.9 mL, 1.04 mol) in phosphoryl chloride (264.2 mL, 2.82 mol) was stirred under microwave irradiation (450 W) at 105 °C for 3 h. After completion of the reaction, the excess of phosphoryl chloride was vacuum evaporated, poured into ice-water, and the resulting solid product was filtered off and dried to afford 162.6 g (93%) of the title compound, which was used for the next step without any further purification. ^1^H NMR (600 MHz, CD_3_OD) δ: 7.42–7.43 (m, 2, H-3, H-5), 6.84 (d, 1, *J* = 8.2 Hz, H-6), 4.80 (s, 2, C*H*_2_). ^13^C NMR (151 MHz, CD_3_OD) δ: 192.23 (*C*OCH_2_), 152.95 (C-3), 146.71 (C-4), 127.87 (C-1), 123.58 (C-6), 116.24 (C-5), 115.99 (C-2), 46.87 (*C*H_2_Cl). (-) ESI QqToF (m/z): Calcd. for C_8_H_6_ClO_3_^−^: [M − H]^−^ 185.0011, found 185.0004.

4-(2-chloroethyl)benzene-1,2-diol (**3**): To a suspension of chloride **2** (80.42 g, 0.43 mol) in trifluoroacetic acid (165.40 mL, 2.15 mol) was added dropwise triethylsilane (276 mL, 2.15 mol) at 0 °C, and the mixture was stirred at room temperature for 11 h. After completion of the reaction, most of the volatiles were removed under reduced pressure, and the oily product was washed with boiling cyclohexane (10 × 500 mL). The cyclohexane layer was decanted and concentrated under reduced pressure to afford 71 g (96%) of compound **3** which was used for the next step without any further purification. M.p.: 102–104 °C (EtOAc/n-hexane). ^1^H NMR (600 MHz, (CD_3_)_2_CO) δ: 7.75 (br s, 2, D_2_O exch., 1-O*H*, 2-O*H*), 6.76 (d, 1, *J* = 8.2 Hz, H-6), 6.75 (s, 1, H-3), 6.60 (dd, 1, *J* = 8.0, 2.2 Hz, H-5), 3.70 (t, 2, J = 7.1 Hz, C*H*_2_Cl), 2.90 (t, 2, *J* = 7.1 Hz, C*H*_2_CH_2_Cl). ^13^C NMR (151 MHz, (CD_3_)_2_CO) δ: 145.80 (C-2), 144.67 (C-1), 130.87 (C-4), 121.06 (C-5), 116.74 (C-6), 116.06 (C-3), 46.10 (*C*H_2_Cl), 39.22 (CH_2_*C*H_2_Cl). (-) ESI QqToF (*m*/*z*): Calcd. for C_8_H_8_ClO_2_^−^: [M − H]^−^ 171.0218, found 171.0224.

Hydroxytyrosol or 4-(2-hydroxyethyl)benzene-1,2-diol (**4**): A suspension of chloride **3** (23 g, 0.13 mol) in distilled water (200 mL) was stirred under microwave irradiation (700 W) at 101 °C for 4 h. After completion of the reaction, the mixture was washed with dichloromethane (2 × 100 mL) and water was removed under reduced pressure or lyophilization to afford 19 g (94.8%) of the title compound as an oil. ^1^H NMR (600 MHz, CD_3_OD) δ: 6.63 (d, 1, *J* = 8.2 Hz, H-6), 6.61 (d, 1, *J* = 2.2 Hz, H-3), 6.48 (dd, 1, *J* = 8.2, 2.2 Hz, H-5), 3.63 (t, 2, *J* = 7.1 Hz, C*H*_2_OH), 2.62 (t, *J* = 7.1 Hz, 2, C*H*_2_CH_2_OH). ^13^C-NMR (151 MHz, CD_3_OD) δ: 146.15 (C-2), 144.62 (C-1), 131.80 (C-4), 121.21 (C-5), 117.07 (C-6), 116.31 (C-3), 64.59 (*C*H_2_OH), 39.65 (*C*H_2_CH_2_OH). (-) ESI QqToF (*m*/*z*): Calcd. for C_8_H_9_O_3_^−^: [M − H]^−^ 153.0557, found 153.0551.

Hydroxytyrosol acetate or 2-(3,4-dihydroxyphenyl)ethyl acetate (**5**): A solution of chloride **3** (12.0 g, 69.2 mmol) and CH_3_COOK (27.23 g, 276.9 mmol) in dry DMF (60 mL) was stirred at 80 °C for 12 h. After completion of the reaction, the excess of DMF was removed under reduced pressure and the crude product was purified by column chromatography (silica gel), eluted by a system of cyclohexane/ethyl acetate 3/1, to afford 11.1 g (82%) of the title compound **5**. M.p.: 81–83 °C (CH_2_Cl_2_/CCl_4_). ^1^H NMR (600 MHz, CDCl_3_) δ: 6.78 (d, 1, *J* = 8.2 Hz, H-5), 6.72 (d, 1, *J* = 2.2 Hz, H-2), 6.59 (dd, 1, *J* = 8.2, 2.2 Hz, H-6), 4.22 (t, 2, *J* = 7.1 Hz, CH_2_O), 2.78 (t, 2, *J* = 7.1 Hz, C*H*_2_CH_2_O), 2.04 (s, 3, COC*H*_3_). ^13^C NMR (151 MHz, CDCl_3_) δ: 172.61 (*C*O), 143.91 (C-3), 142.61 (C-4), 130.32 (C-1), 121.25 (C-6), 116.01 (C-5), 115.55 (C-2), 65.71 (*C*H_2_O), 34.36 (*C*H_2_CH_2_O), 21.12 (*C*H_3_). (-) ESI QqToF (m/z): Calcd. for C_10_H_11_O_4_^−^: [M − H]^−^ 195.0663, found 195.0655.

1-(3,4-dihydroxyphenyl)-2-hydroxyethanone (**6**): This compound was synthesized with an analogous procedure as described for compound **4**, starting from the chloride **2**, in 91% yield. ^1^H NMR (600 MHz, (CD_3_)_2_CO) δ: 7.48 (d, 1, *J* = 2.2 Hz, H-2), 7.44 (dd, 1, *J* = 8.2, 2.2 Hz, H-6), 6.94 (d, 1, *J* = 8.2 Hz, H-5), 4.76 (s, 2, C*H*_2_). ^13^C NMR (151 MHz, (CD_3_)_2_CO) δ: 197.81 (*C*O), 151.61 (C-3), 145.95 (C-4), 127.54 (C-1), 122.16 (C-6), 115.94 (C-5), 115.28 (C-2), 65.53 (*C*H_2_). (-) ESI QqToF (m/z): Calcd. for C_8_H_7_O_4_^−^: [M − H]^−^ 167.0350, found 167.0357.

3,4-dihydroxyphenylglycol (DHPG, **7**): A solution of **6** (1.4 g, 8.33 mmol) in methanol (80 mL) was hydrogenated in the presence of 10% Pd/C (100 mg), under a pressure of 50 psi, at room temperature for 9 h. The resulting mixture was filtered through a Celite pad, and the filtrate was evaporated to dryness. Treatment with activated charcoal in hot methanol and filtration through a Celite pad afforded the title compound practically pure (1.13 mg, 80%). For analytical reasons, a small amount was quickly purified (due to stability reasons) by column chromatography (silica gel) using a mixture of cyclohexane/ethyl acetate 1/4 as the eluent. ^1^H NMR (600 MHz, CD_3_OD) δ: 6.83 (d, 1, *J* = 2.0 Hz, H-2), 6.74 (d, 1, *J* = 8.2 Hz, H-5), 6.68 (d, 1, *J* = 8.2 Hz, H-6), 4.55 (t, 1, *J* = 6.1 Hz, C*H*CH_2_), 3.58 (d, 2, *J* = 6.0 Hz, CHC*H*_2_). ^13^C NMR (151 MHz, CD_3_OD) δ: 146.07 (C-3), 145.72 (C-4), 134.72 (C-1), 118.98 (C-6), 116.11 (C-5), 114.64 (C-2), 75.70 (*C*HCH_2_), 68.69 (CH*C*H_2_). (-) ESI QqToF (*m*/*z*): Calcd. for C_8_H_9_O_4_^−^: [M − H]^−^ 169.0506, found 169.0512.

4-(1-ethoxy-2-hydroxyethyl)benzene-1,2-diol (**8**): To a solution of **6** (200 mg, 1.19 mmol) in abs. ethanol (20 mL) was added HClO_4_ (100 μL) and the mixture was hydrogenated in the presence of 10% Pd/C (20 mg), under a pressure of 50 psi, at room temperature, for 4 h. The resulting mixture was filtered through a Celite pad and the filtrate was evaporated to dryness and the residue was purified by column chromatography (silica gel) using a mixture of cyclohexane/ethyl acetate 1/2 as the eluent to afford 198 mg (84%) of the title compound. ^1^H NMR (600 MHz, (CD_3_)_2_CO) δ: 7.81 (br s, 2, D_2_O exch., 1-O*H*, 2-O*H*, 6.81 (d, 1, *J* = 2.2 Hz, H-3), 6.78 (d, 1, *J* = 8.2 Hz, H-6), 6.65 (dd, 1, *J* = 8.2, 2.2 Hz, H-5), 4.20 (m, 1, C*H*CH_2_), 3.54–3.60 (m, 1, CHC*H*_2_OH), 3.39–3.47 (m, 2, CHC*H*_2_OH, C*H*_2_CH_3_), 3.32–3.38 (m, 1, C*H*_2_CH_3_), 1.12 (t, 3, *J* = 7.1 Hz, CH_2_C*H*_3_). ^13^C NMR (151 MHz, (CD_3_)_2_CO) δ: 145.83 (C-2), 145.44 (C-1), 132.74 (C-4), 119.38 (C-5), 115.85 (C-6), 114.67 (C-3), 83.75 (*C*HCH_2_), 67.96 (CH*C*H_2_OH), 64.58 (*C*H_2_CH_3_), 15.65 (CH_2_*C*H_3_). (-) ESI QqToF (m/z): Calcd. for C_10_H_13_O_4_^−^: [M − H]^−^ 197.0819, found 197.0811.

## 4. Conclusions

In summary, a new, large-scale, straightforward synthetic approach for the preparation of HT as well as HT acetate and DHPG has been described in the present work, using simple, low-cost, and scalable procedures, starting from inexpensive, commercially available catechol. The established procedure for the synthesis of HT acetate could be used for the synthesis of other lipophilic esters of HT. Additionally, a synthetic approach for the synthesis of compound **8** has been proposed, which could be used for the development of new lipophilic and stable analogs of DHPG. All the reactions proceeded smoothly, with no byproducts formed, while the purification of the compounds was very simple, mostly by solvent trituration. The synthetic procedures were based in microwave irradiation, which enabled more precise control of the reaction conditions and contributed to higher yields, thus, the new synthetic approaches could be an alternative for the industrial preparation of HT, HT acetate and DHPG.

## Figures and Tables

**Table 1:** Comparison of the methodologies for the preparation of hydroxytyrosol (HT), HT Acetate and 3,4-dihydroxyphenylglycol (DHPG).

A/A	Reference ^a^	Starting Material	Short Description	Steps	Overall Yield (%)
Synthesis of Hydroxytyrosol					
1	This Work	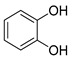		3	85
2	WO2007/009590A1 [63]		Synthesis via preparation of 3,4-dihydroxymandelic acid and reduction with Pd/C to afford 3,4-dihydroxyphenylacetic acid, which upon reduction with LiAlH_4_ resulted to HT.	4	57
3	WO2008/107109A1 [54]		Synthesis through chloroacetylation and reduction over Pd/C.	3	56
4	Chem. Sus. Chem. 2018, 11, 2202–2210 [53]		Synthesis through preparation of 3,4-dihydroxymandelic acid and hydrogenation with Pd/C.	2	30
5	Synth. Commun. 2007, 37, 4245–4252 [58]	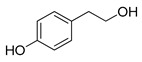	Synthesis through oxidative bromination of tyrosol and subsequent methoxylation/deprotection.	6	67
6	Bioorg. Chem. 2007, 35, 137–152 [69]		Enzymatic synthesis with tyrosinase.	1	95
7	WO2008/110908A1 [64]		Selective oxidative hydroxylation of protected tyrosol by Dess Martin reagent followed by deprotection.	3	40
8	J. Agric. Food Chem. 2008, 56, 8897–8904 [57]		Selective oxidative hydroxylation of protected tyrosol by Dess Martin periodinane.	3	67
9	Tetrahedron Lett. 2009, 50, 1307–1310 [62]		Selective oxidative hydroxylation of protected tyrosol by polymer-supported 2-iodoxybenzoic acid.	1	quant.
10	US2009/0023815A1 [51]		Enzymatic hydroxylation of tyrosol with tyrosinase.	1	20
11	Tetrahedron Lett. 2011, 52, 4938–4940 [59]		Synthesis through selective formylation and Baeyer–Villiger oxidation.	4	59
12	J. Agric. Food Chem. 1999, 47, 1745–1748 [48]	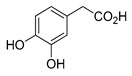	Reduction via LiAlH_4_	1	79
13	J. Agric. Food Chem. 2000, 48, 4087–4090 [60]		Reduction via LiAlH_4_ or tetrabutylammonium boronate	1	45
14	Molecules, 2007, 12, 1762–1770 [52]		Reduction of protected 3,4-dihydroxyphenylacetic acid via LiAlH_4_ and deprotection via Amberlyst 15.	1	80
15	WO2017/149017A1 [66]	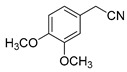	Preparation through deprotection, hydrolysis, and reduction of the resulting acid via LiAlH_4_.	4	36
16	WO2012/003625A1 [65]	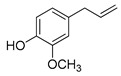	Preparation through ozonolysis and deprotection via AlCl_3_/EtSH system.	3	75
17	J. Agric. Food Chem. 2007, 55, 3386–3391 [50]	Oleuropein	Preparation through basic hydrolysis of protected oleuropein and deprotection via Amberlyst 15.	1	11
18	Mediterr. J. Chem. 2018, 7, 217–222 [49]	Oleuropein (pure)	Preparation through acidic hydrolysis of oleuropein obtained from *Ligustrum Vulgare*.	1	73
Synthesis of Hydroxytyrosol Acetate					
1	This Work	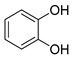		3	73
2	Synth. Commun. 2007, 37, 4245–4252 [58]	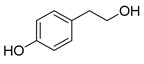	Synthesis through oxidative bromination of tyrosol and subsequent methoxylation, bis-acetylation and selective deprotection.	5	70
3	WO2008/110908A1 [64]		Selective oxidative hydroxylation of tyrosol acetate by Dess Martin reagent.	3	40
4	J. Agric. Food Chem. 2008, 56, 8897–8904 [57]		Selective oxidative hydroxylation of tyrosol acetate by Dess Martin periodinane.	2	76
5	Tetrahedron Lett. 2011, 52, 4938–4940 [59]		Synthesis through selective formylation and Baeyer–Villiger oxidation of tyrosol. acetate	4	66
6	Bioorg. Med. Chem. 2015, 23, 5345–5351 [68]		Enzymatic hydroxylation of tyrosol with tyrosinase subsequent esterification via *Candida antarctica* lipase.	1	98
7	J. Agric. Food Chem. 2001, 49, 2480–2485 [38]	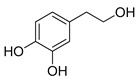	Synthesis through dibenzyloxy-HT, acetylation and subsequent deprotection.	3	25
8	US2003/0236202 [73]		Selective esterification with K_2_CO_3_ and tetrabutylammonium hydrogen sulfate.	1	57
9	WO2004/005237A1 [71]		Selective transesterification via ethylacetate and p-toluenesulfonic acid.	1	86
10	EP1541544A1 [72]		Enzymatic esterification with porcine pancreatic lipase.	1	86
Synthesis of DHPG					
1	This Work	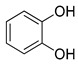		3	70
2	J. Med. Chem. 1963, 607–608 [74]	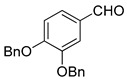	Synthesis via hydrolysis of 3,4-dibenzyloxymandelonitrile, reduction of the resulting acid via LiAlH_4_ and deprotection via Pd/C.	4	20

^a^ Each number in the brackets represents the number in the reference section.

## Supplementary Materials

^1^H and ^13^C-NMR spectra are available online.
