# Supplementary material for: A Three-Step, Gram-Scale Synthesis of Hydroxytyrosol, Hydroxytyrosol Acetate, and 3,4-Dihydroxyphenylglycol
Source: Molecules. 2019 Sep 5;24(18):3239. doi: 10.3390/molecules24183239 (PMC6767028; doi:10.3390/molecules24183239)
Supplement: Supplementary file 1 [file molecules-24-03239-s001.pdf]

molecules

MDPI

Article

A three-step, gram-scale synthesis of Hydroxytyrosol, Hydroxytyrosol Acetate, and 3,4-dihydroxyphenylglycol.

Amalia D. Kalampaliki[1], Vassiliki Giannouli[1], Alexios-Leandros Skaltsounis[2], Ioannis K. Kostakis[1*].

[1] *Division of Pharmaceutical Chemistry, Department of Pharmacy, National and Kapodistrian University of Athens, Panepistimiopolis-Zografou, Athens 15771, Greece*

[2] *Division of Pharmacognosy & Natural Product Chemistry, Department of Pharmacy, National and Kapodistrian University of Athens, Panepistimiopolis-Zografou, Athens 15771, Greece*

* Correspondence: ikkostakis@pharm.uoa.gr

Contents

Figure S1: ^{1}H-NMR and ^{13}C NMR spectrum of compound **2**.

Figure S2: ^{1}H-NMR and ^{13}C NMR spectrum of compound **3**.

Figure S3: ^{1}H-NMR and ^{13}C NMR spectrum of compound **4**.

Figure S4: ^{1}H-NMR and ^{13}C NMR spectrum of compound **5**.

Figure S5: ^{1}H-NMR and ^{13}C NMR spectrum of compound **6**.

Figure S6: ^{1}H-NMR and ^{13}C NMR spectrum of compound **7**.

Figure S7: ^{1}H-NMR and ^{13}C NMR spectrum of compound **8**.

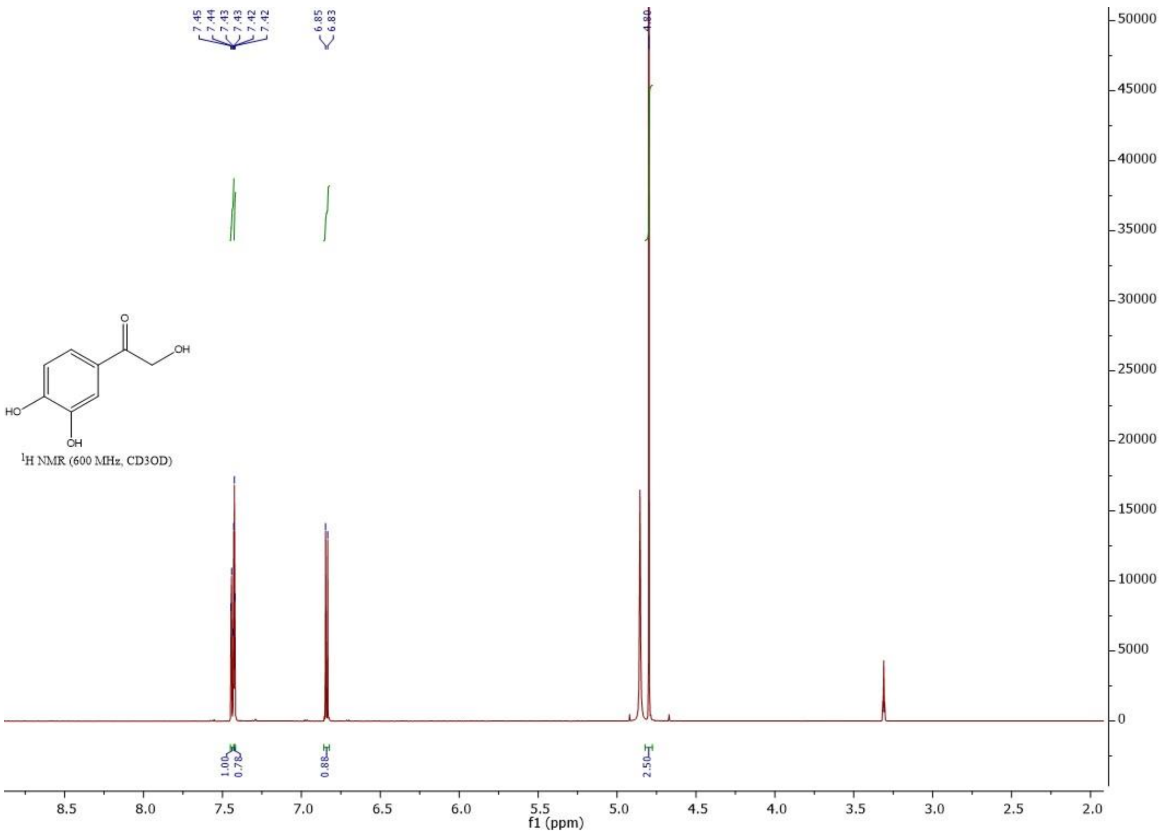

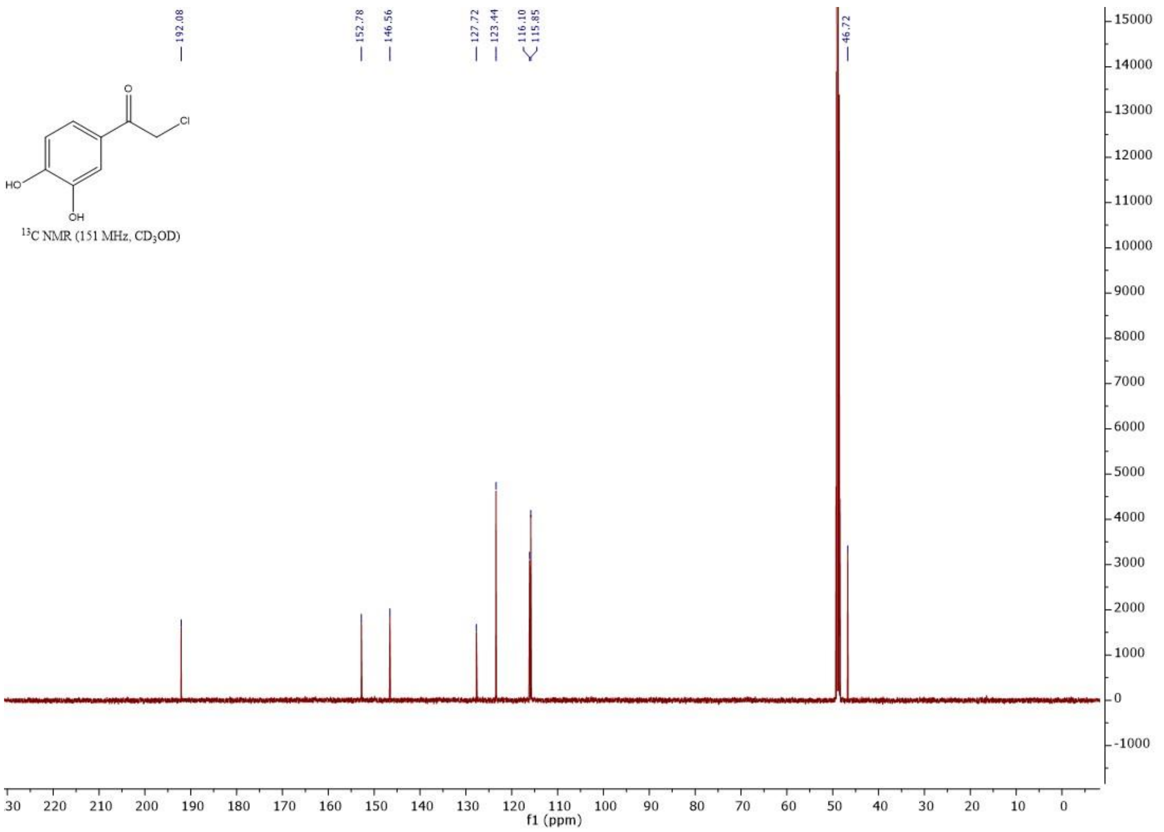

Figure S1: ^{1}H-NMR and ^{13}C NMR spectrum of compound **2**.

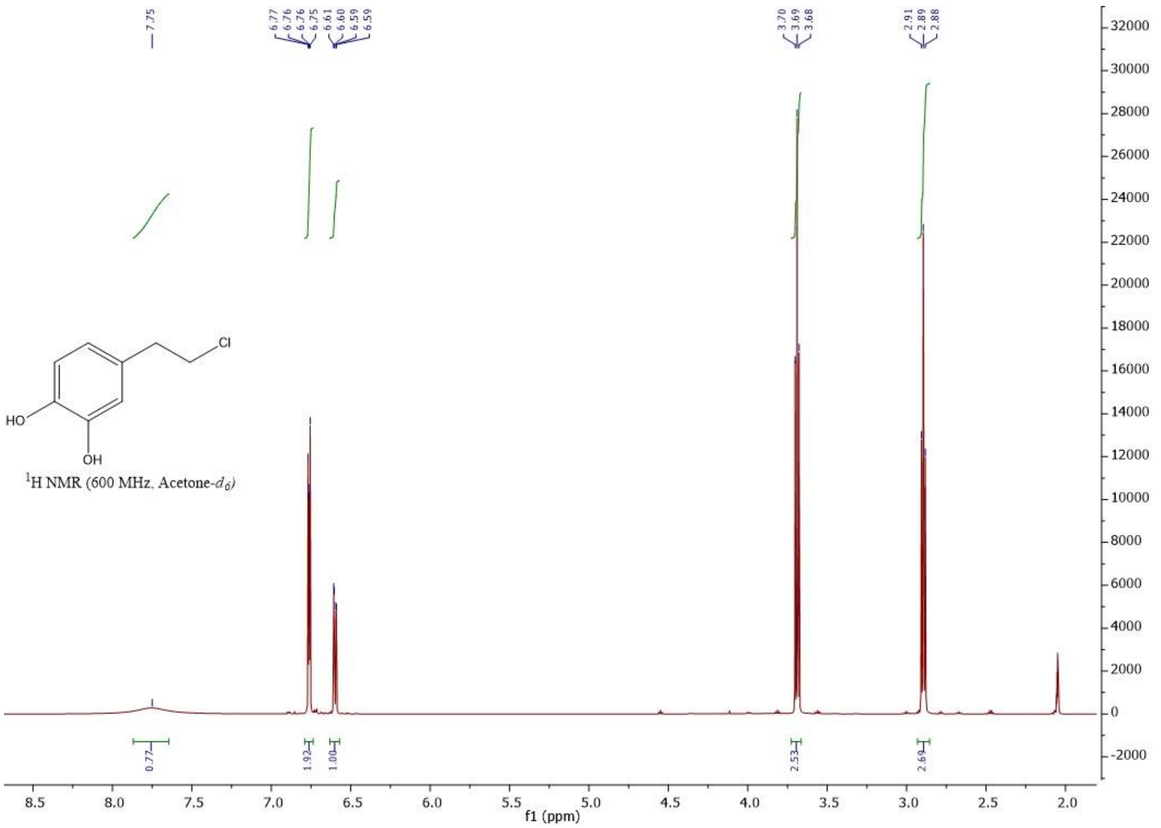

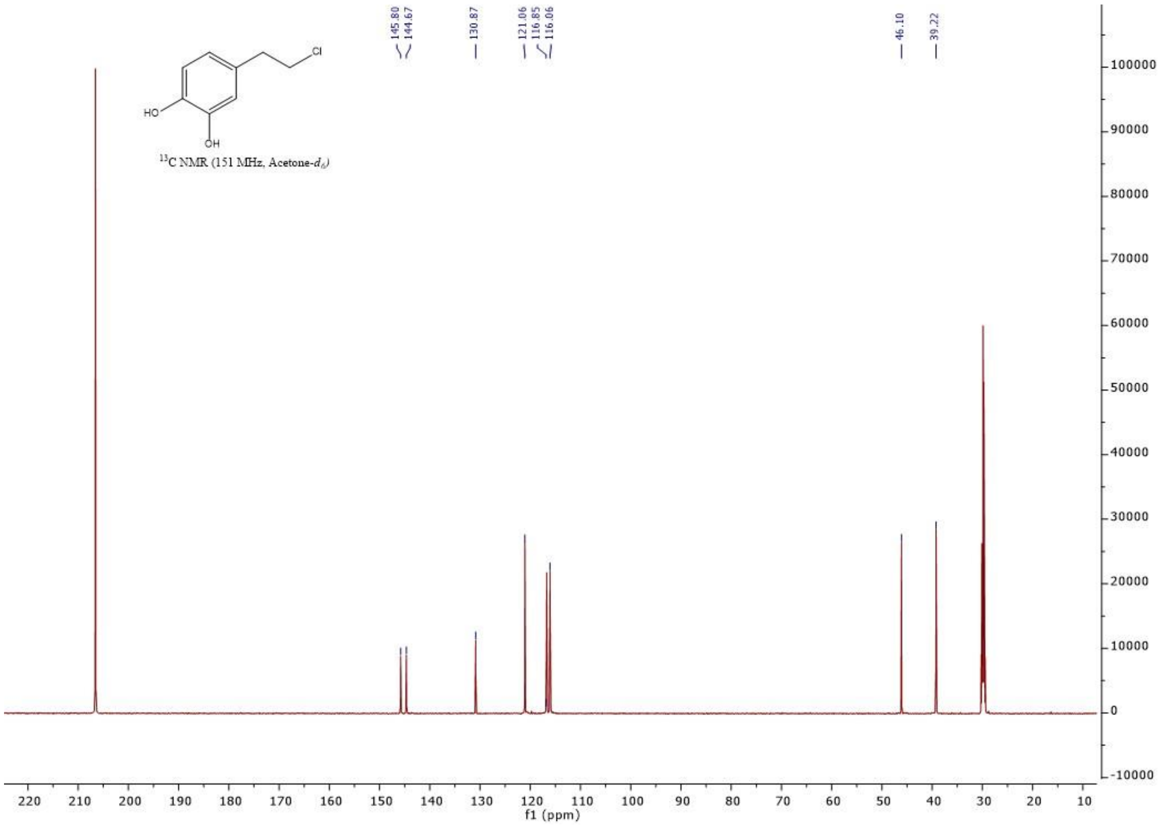

Figure S2: ^{1}H-NMR and ^{13}C NMR spectrum of compound **3**.

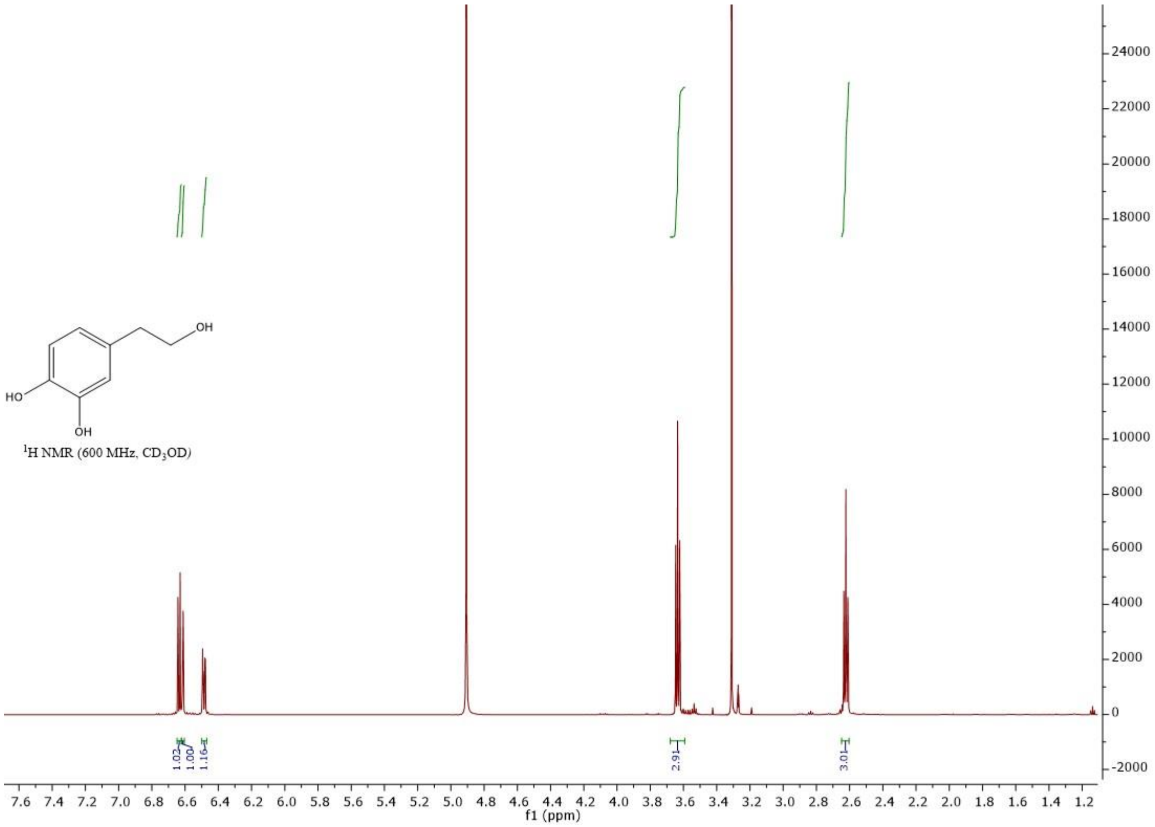

Figure S3: ^{1}H-NMR and ^{13}C NMR spectrum of compound **4**.

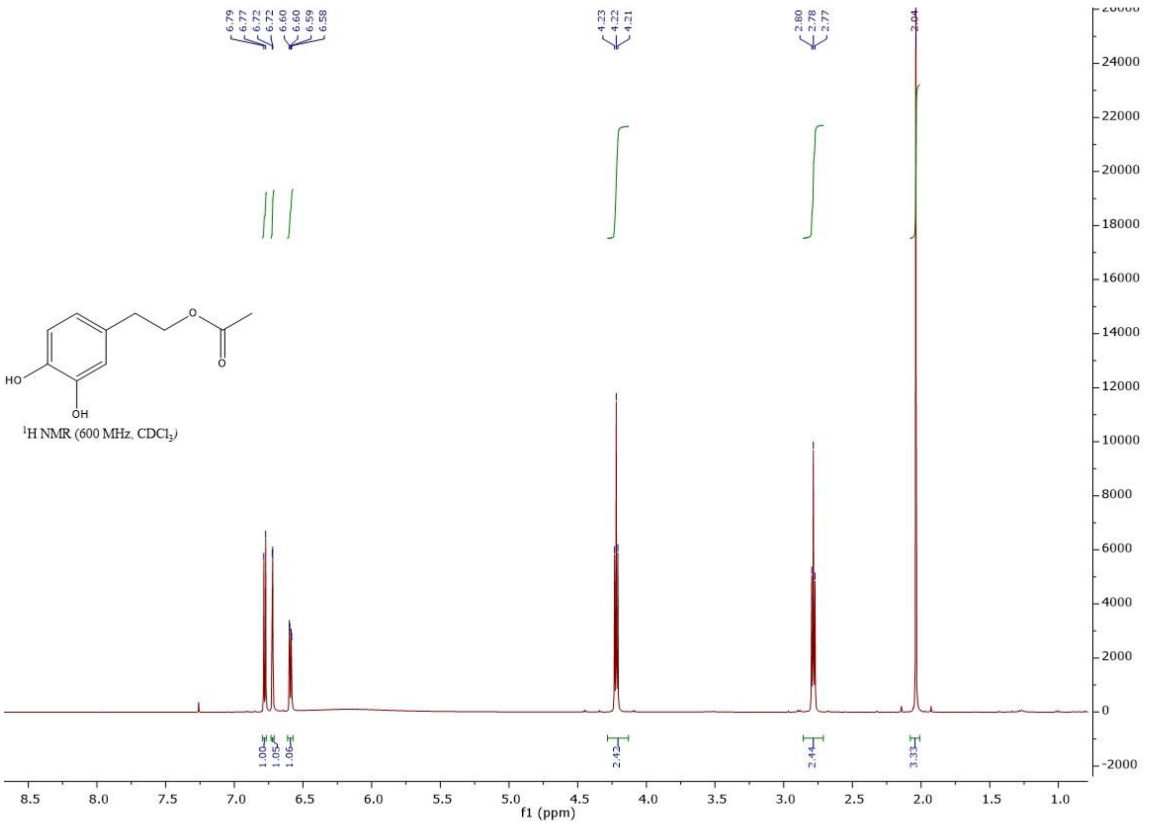

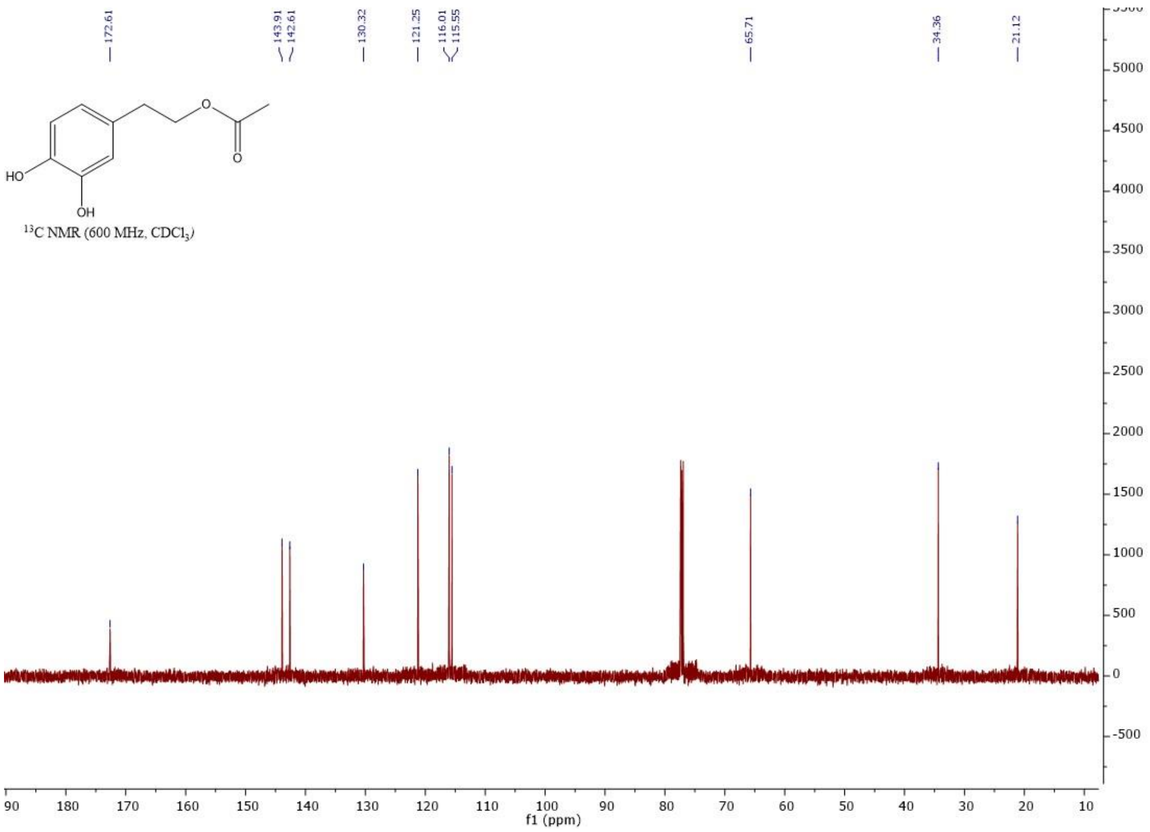

Figure S4: ^{1}H-NMR and ^{13}C NMR spectrum of compound **5**.

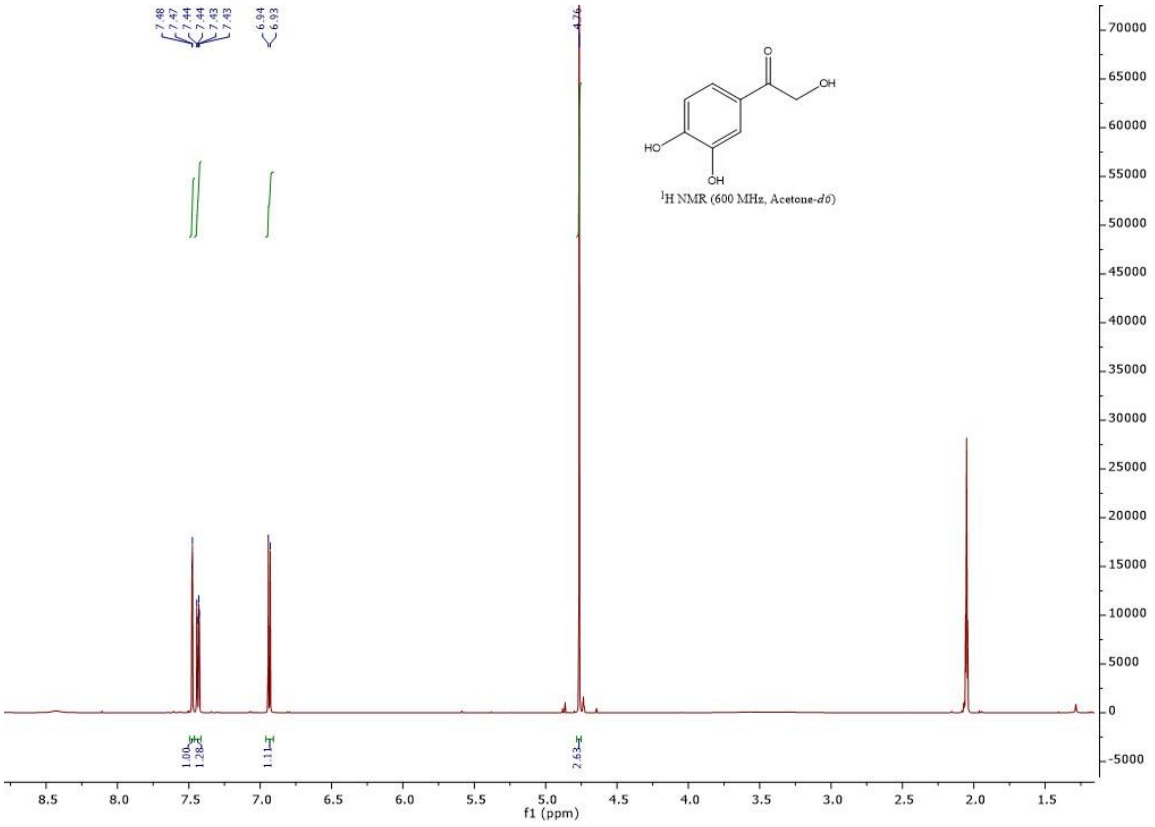

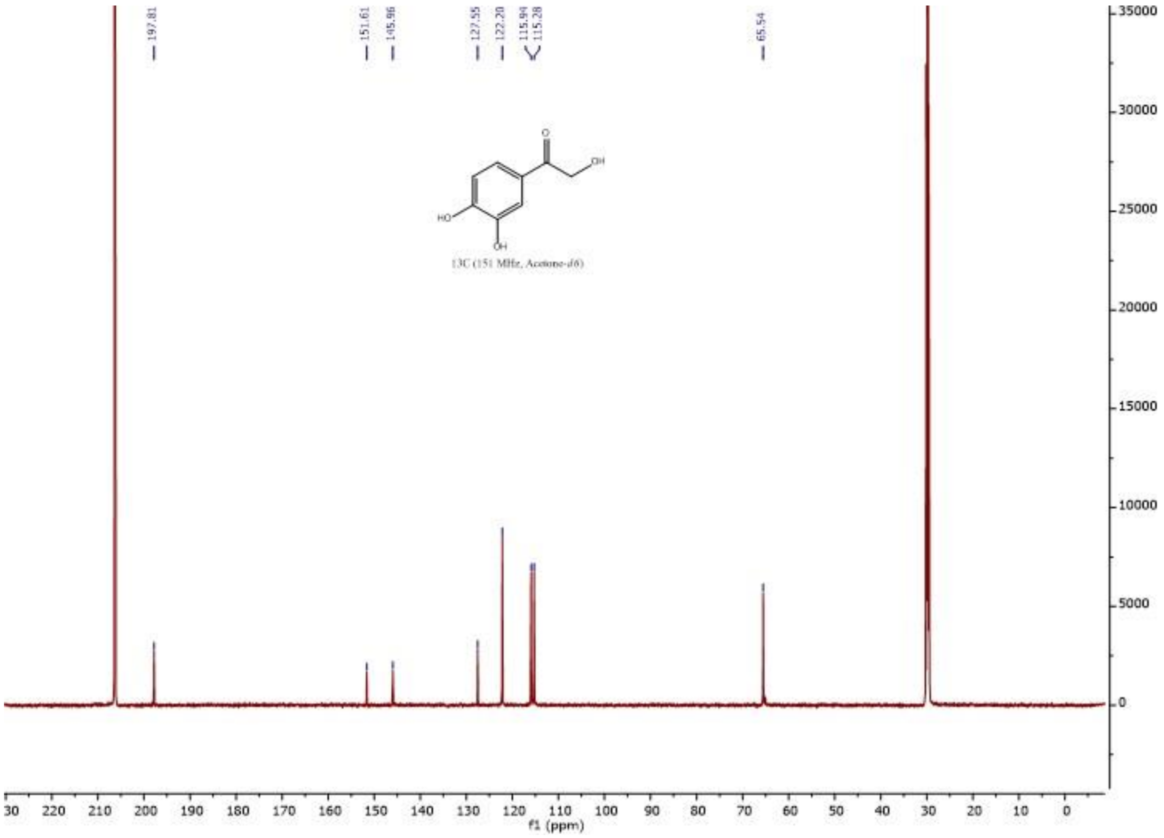

Figure S5: ^{1}H-NMR and ^{13}C NMR spectrum of compound **6**.

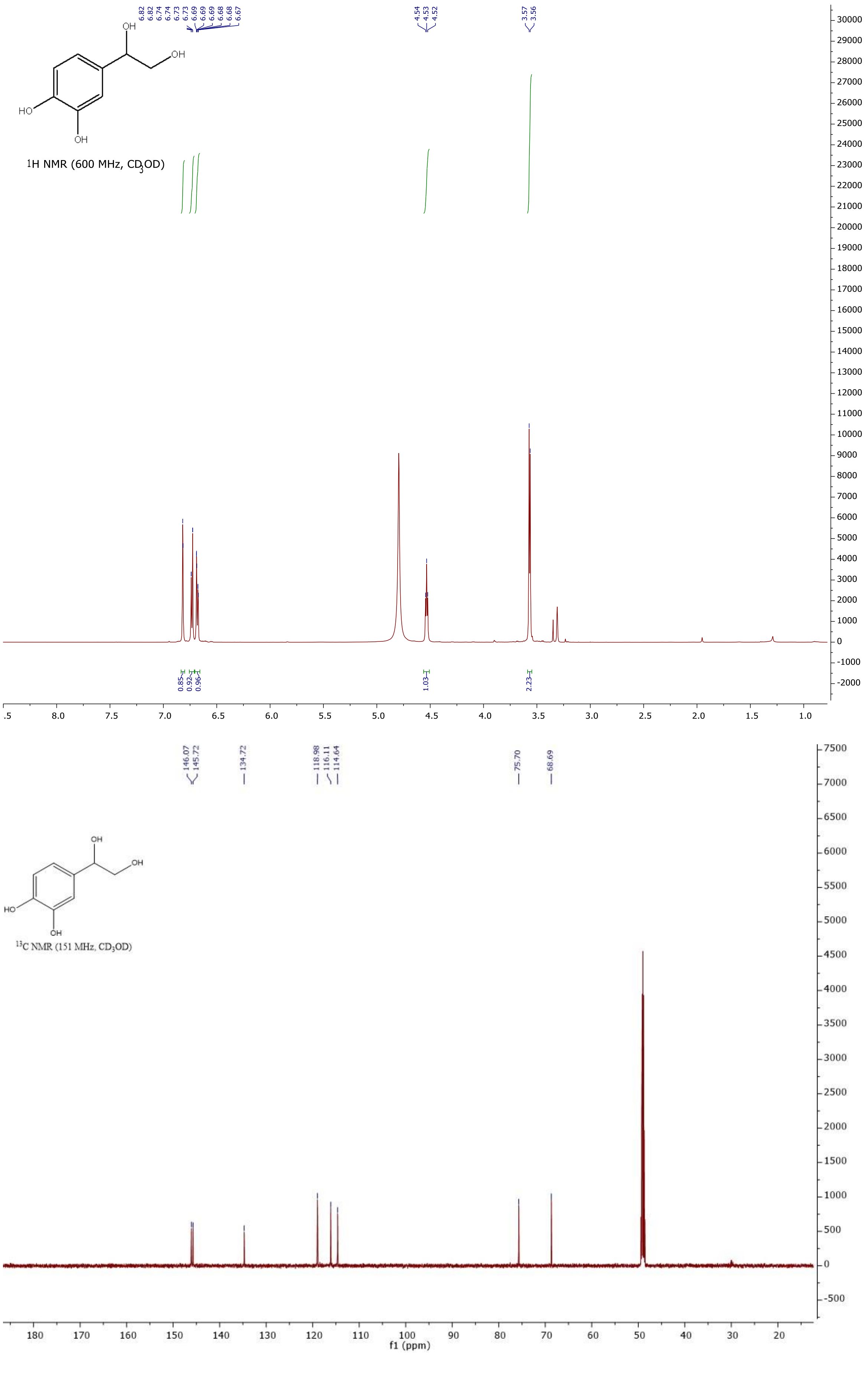

Figure S6: ^{1}H-NMR and ^{13}C NMR spectrum of compound **7**.

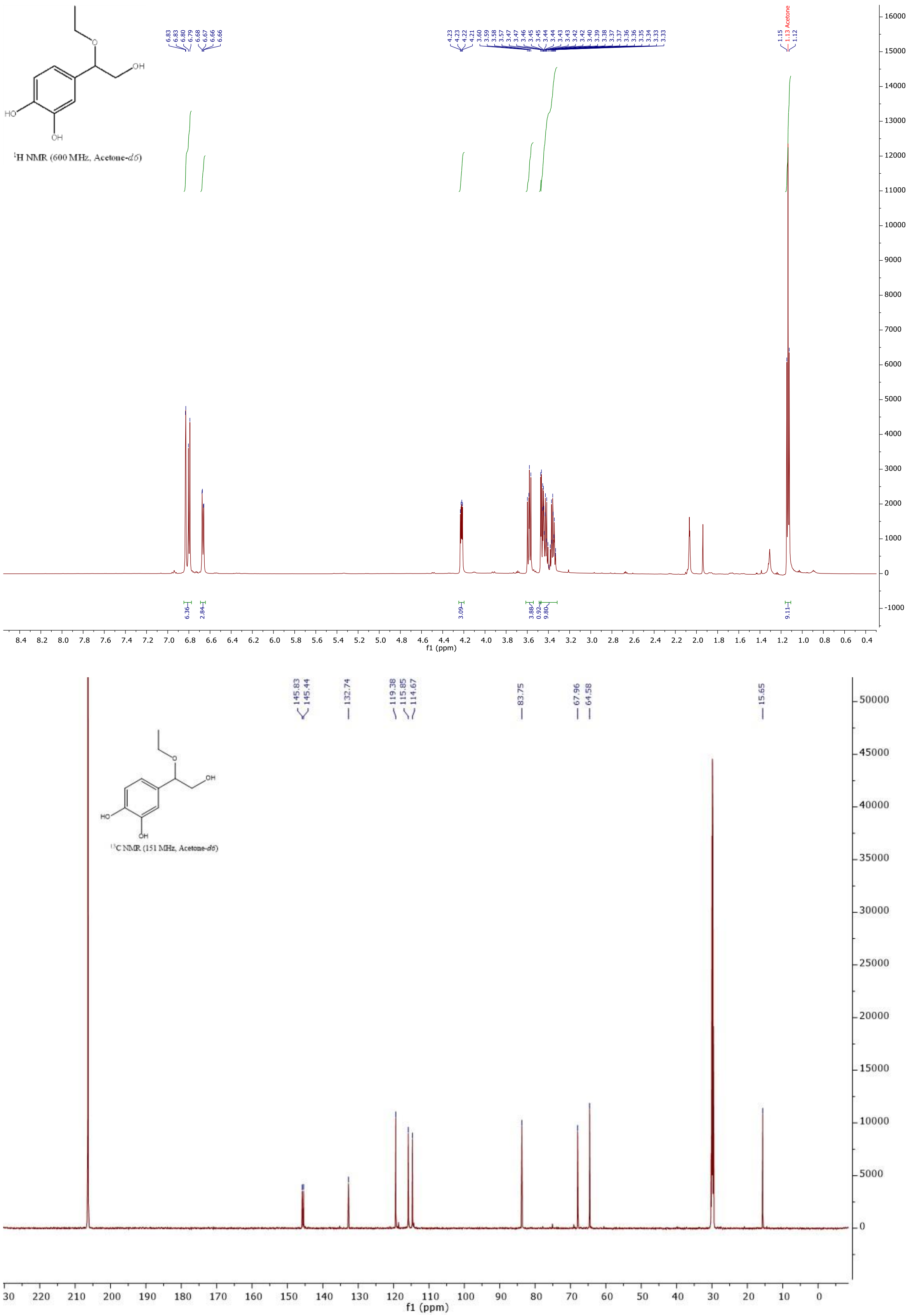

Figure S7: ¹H-NMR and ¹³C NMR spectrum of compound **8**.
